# Ubiquitylation Extends to Lipid Substrate for Restricting Bacterial Infection

**DOI:** 10.3389/fmolb.2021.791009

**Published:** 2021-11-22

**Authors:** Chaofeng Wang, Lirong Zeng

**Affiliations:** Department of Plant Pathology, Center for Plant Science Innovation, University of Nebraska, Lincoln, NE, United States

**Keywords:** ubiquitylation (ubiquitination), lipopolysaccharide (LPS), intracellular bacteria, immune signaling, Rnf213, posttranslational modification

The substrates of ubiquitylation have been recognized as protein ever since the ubiquitin modifier was discovered decades ago. However, this conventional perception is fundamentally changed by a recent finding that lipopolysaccharide (LPS) molecules are ubiquitylated by host cells to trigger cell-autonomous immunity against bacterial invasion.

Ubiquitylation (or ubiquitination) has been documented to be of vital importance in virtually all cellular and physiological pathways. The ubiquitylation process is accomplished through a stepwise enzymatic cascade typically catalyzed by three different enzymes: E1 (ubiquitin-activating enzyme), E2 (ubiquitin-conjugating enzyme), and E3 (ubiquitin ligase). The sequential action of the E1–E2–E3 cascade results in covalent attachment of ubiquitin to the selected substrates. Until very recently, ubiquitylation has been recognized as an exclusive post-translational protein modification. However, a recent landmark study by Otten and colleagues revealed that host cells combat invading bacteria in the cytosol by tagging the lipid A moiety of bacterial lipopolysaccharide (LPS) with ubiquitin, which leads to subsequent autophagic degradation of the pathogens (Otten et al., 2021).

In general, intracellular bacterial pathogens such as *Salmonella* enter host cells by receptor-mediated endocytosis followed by forming a membrane-bound vacuolar compartment termed bacteria-containing vacuole (BCV), which further develops into a replicative niche. To propagate and disseminate, bacteria inside the BCV need access the host cytosol and finish a cytoplasmic lifestyle through vacuolar rupture (Santos and Enninga, 2016; Steele et al., 2016) (Figure 1). Although vacuolar rupture promotes cytosolic multiplication, damage of BCV exposes the pathogens to the surveillance of host innate immune system, leading to coating the bacteria with ubiquitin and subsequent activation of antibacterial autophagy (Perrin et al., 2004). Due to importance of the ubiquitylation system in host immune response to pathogen infection, extensive efforts have been made to identify components involved in the ubiquitylation of cytosolic bacteria (Tripathi-Giesgen et al., 2021). Nevertheless, the machinery and molecular mechanism that earmark the intracellular bacteria with host ubiquitin remain largely unknown.

**FIGURE 1:**
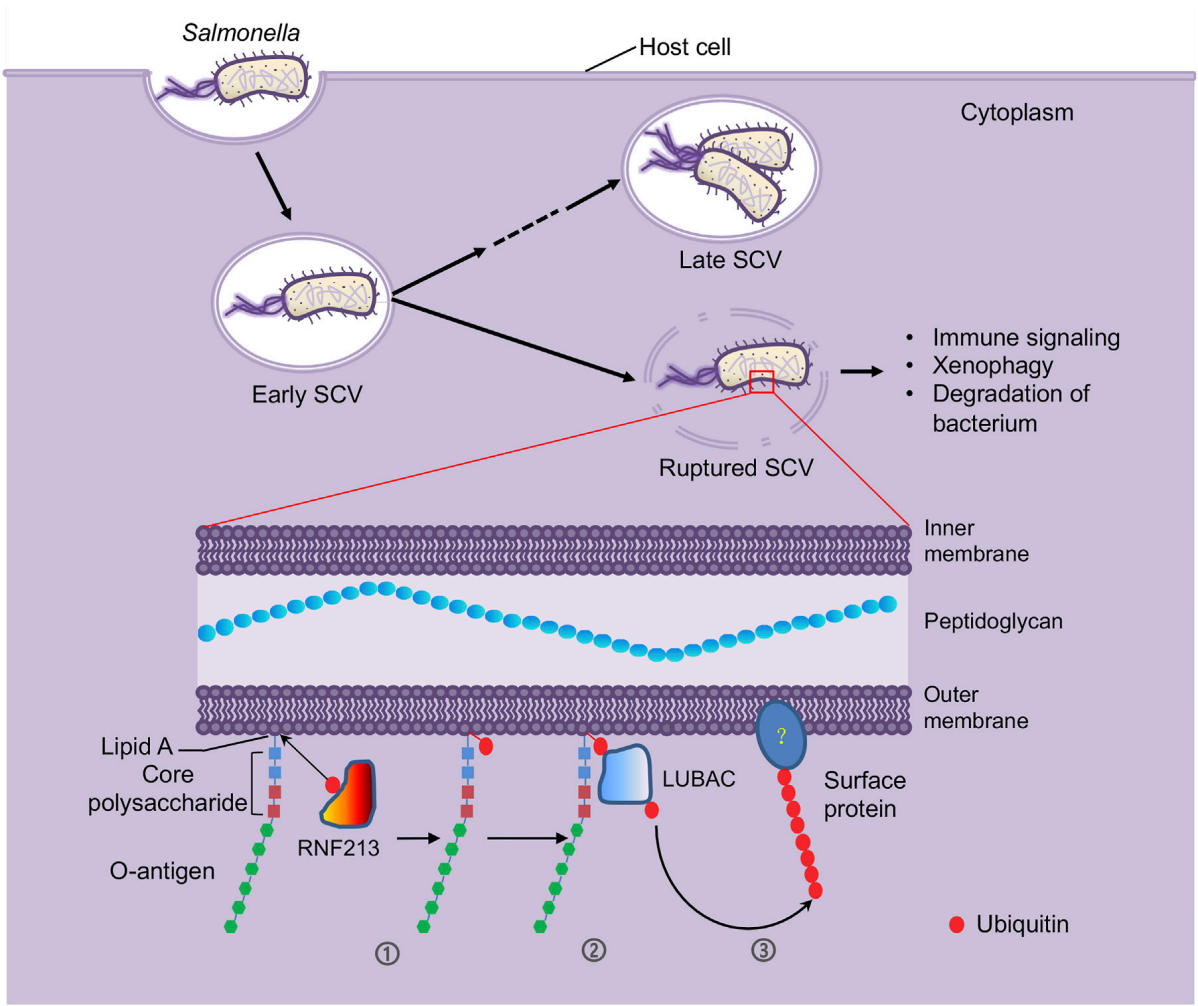
LPS molecules are ubiquitylated by host E3 enzyme RNF213 as a defense mechanism against intracellular bacterial infection. After invasion into host cells by forming a membrane-bound vacuole called *Salmonella*-containing vacuole (SCV), *S*. Typhimurium bacteria adopt a bimodal lifestyle, replicating within the vacuolar compartments as well as in the host cytosol by inducing membrane lysis of the vacuoles. However, the vacuole rupture can expose the invading bacteria to host ubiquitin system (Tripathi-Giesgen et al., 2021), where the host E3 ligase RNF213 ubiquitylates the LPS molecules. LPS is a unique component of the outer monolayer of the outer membranes of the Gram-negative bacteria, comprised of lipid A, core polysaccharides and O-antigen repeats, of which lipid A is recognized and ubiquitylated by RNF213 (➀). RNF213-mediated LPS ubiquitylation serves as a pattern recognition receptor to recruit another host E3 ligase LUBAC (➁). The LUBAC then catalyzes formation of linear ubiquitin chain on a yet unidentified substrate (➂). These ubiquitylation events initiate immune signaling and xenophagy, which results in bacterium clearance.

Otten and colleagues strived to fill the knowledge gap by decoding the ubiquitin coat of *Salmonella enterica* serovar Typhimurium (*S*. Typhimurium) in human cells (Otten et al., 2021). They first monitored the dynamic localization of the ubiquitin coat using structured illumination microscopy and found the ubiquitin signals were localized on the surface of *S*. Typhimurium that escaped from the invasion vacuoles at 4 h after infection (HAI). The authors then prepared wild type and O-antigen polymerase defective mutant of *S*. Typhimurium from infected cells at 4 HAI for analysis of the ubiquitin coat formation, which led to the discovery that the ubiquitylated substrate were LPS molecules instead of proteins. The finding was further supported by the observation that the low molecular weight ubiquitin bands in the O-antigen polymerase defective mutant of *S*. Typhimurium and the majority of ubiquitin smear in wild type bacteria are heat resistant (i.e., soluble after boiling).

LPS is a group of structurally related glycolipids constituting the outer leaflet of the outer membrane of most Gram-negative bacteria. LPS molecules show a typically tripartite structure: a hydrophobic domain known as lipid A, a short “core” oligosaccharide, and a long-chain, repeat-unit polysaccharide (O-antigen repeats) (Whitfield et al., 2020) (Figure 1). Many key genes dedicated to LPS biosynthesis have been identified and the length and molecular weight of LPS in mutants of these genes are altered. Immunoblotting with samples purified from *Salmonella* mutants defective in the biosynthesis of the core polysaccharides and O-antigen indicated that changes in the ubiquitylation patterns match the alterations in LPS length and molecular weight, which further confirmed LPS is the target of ubiquitylation on cytosolic *S*. Typhimurium.

Ubiquitin molecules are known to append to the protein targets through isopeptide bonds with amino groups (amide-linkage) or occasionally through ester bonds with hydroxy groups (ester-linkage). The authors tested whether amino groups that can be introduced onto LPS by numerous transferases are essential for LPS ubiquitylation by deleting genes that encode the transferases. They found loss of function in either one or all the genes exerted no impairments to LPS ubiquitylation, which excluded the known amino groups in LPS as sites of ubiquitylation. To examine the possibility of ester-linked ubiquitin, the authors treated O-antigen polymerase defective mutant of *S*. Typhimurium isolated from the infected host cells under alkaline conditions and found the treatment resulted in hydrolysis of the ubiquitin chains attached to LPS. Thus, ubiquitin molecules attach to LPS through ester-linkage.

To pinpoint the E3 enzyme responsible for the ubiquitylation of LPS, Otten and colleagues executed a procedure of extensive fractionation and purification of the HeLa cell lysates and tested each fraction for the E3 activity that is responsible for LPS ubiquitylation, leading to the identification of RNF213 (Figure 1), an E3 that is known to be connected to the moyamoya disease. Knocking down and knocking out *RNF213* by siRNA and CRISPR technology, respectively, revealed that lack of RNF213 abolished LPS ubiquitylation on cytosolic *S. Typhimurium* in both human and mouse cells. *In vitro* ubiquitylation reaction found that purified RNF213 ubiquitylated LPS in a classical manner where ATP and E1 and E2 enzymes are required. When lipid A alone and various truncated versions of LPS purified from *Salmonella enterica* ser. Minnesota strains were used as substrate of the *in vitro* reaction, lipid A was found to be the minimal form of LPS being ubiquitylated by RNF213.

RNF213 is a giant, multi-domain E3 enzyme that contains a long-disordered region followed by a stalk at the N-terminal, a dynein-like ATPase core in the middle, and a RING-type E3 ligase module at the C-terminal (Ahel et al., 2020). Genetic complementation using various truncations and point mutations of *RNF213* showed the dynein-like ATPase core was indispensable for LPS ubiquitylation. Surprisingly, the RING domain was not required for the ubiquitylation of LPS by RNF213. Neither did the mutations in RNF213 that are associated with the moyamoya disease patients cause defect in LPS ubiquitylation. Instead, a C-terminal RNF213 region that is adjacent to the RING domain and embedded with a conserved 27-amino-acid stretch consisting of four cysteine (C) and two histidine (H) amino acid residues (CHC3H) named by the authors as RZ finger (RNF213–ZNFX1 finger) (Wang et al., 2019) is essential for RNF213-mediated auto-ubiquitylation and ubiquitylation of LPS.

The ubiquitin coat associated with cytosolic bacteria serves as a signal for the xenophagy pathway, which plays a key role in cell-autonomous immunity. Microscopy studies showed that RNF213 colocalized with the ubiquitin coat by forming a layer of RNF213 surrounding the cytosol-invading *S*. Typhimurium and the dynein-like AAA + module and E3 ligase activity were indispensable for generating a stable RNF213 coat. The authors observed that RNF213-deficient mouse embryonic fibroblast (MEF) cells couldn’t produce a ubiquitin coat surrounding the *S*. Typhimurium cells. The RNF213-deficient MEF cells also failed to recruit the ubiquitin interacting autophagy cargo receptors thus caused an impairment in antibacterial autophagy and presented an increased proliferation of the intracellular *S*. Typhimurium. The authors thus concluded that RNF213 plays a positive role in host immune response to bacterial infection in a ubiquitin-dependent manner.

Another E3 ligase, LUBAC (linear ubiquitin chain assembly complex) that catalyzes formation of Met1-linked linear ubiquitin chains was previously shown to contribute to cell-autonomous immunity and requires pre-existing ubiquitin for its own recruitment (Figure 1) (Noad et al., 2017). Otten and colleagues found that lack of RNF213 impaired the recruitment of LUBAC to and formation of Met1-linked ubiquitin chains on the surface of the cytosolic *S*. Typhimurium, indicating that RNF213-mediated LPS ubiquitylation functions to initiate LUBAC-mediated immune signaling. To date, evidence for the involvement of LUBAC in host immunity against intracellular Gram-positive bacteria is very limited. A recent work revealed that RNF213 also facilitates restricting the growth of *Listeria monocytogenes* in an E3 ligase activity-dependent but ubiquitylation-independent manner (Thery et al., 2021), which prompts the question of whether LUBAC is involved in RNF213-facilitated host immunity against Gram-positive bacterial pathogens.

During coevolution with their hosts, bacterial pathogens have evolved multiple strategies to escape or hijack components of the host ubiquitin machinery, one of which is through deubiquitylation. In consistence, the ubiquitylation of LPS was found to be antagonized by the deubiquitylating enzyme (DUB) USP2 *in vitro*, raising the possibility that *S*. Typhimurium may utilize DUBs derived from the host or synthesized and secreted by the pathogen to remove ubiquitin from LPS for promoting infections. OTULIN, a well-known Met1-specific host DUB was recently shown to function at the host–*Salmonella* interface and down-regulates NF-κB signaling by restricting Met1-ubiquitin chains formation (Van Wijk et al., 2017). In addition, two effector proteins from *S*. Typhimurium, SseL and AvrA are translocated into the host cells during infection where they facilitate virulence and survival of bacteria by preventing NF-κB activation and protecting the *Salmonella*-containing vacuole (SCV) stability through their deubiquitylating activity (Le Negrate et al., 2008; Kolodziejek et al., 2019). However, whether SseL, AvrA or other unidentified bacterial effectors can reverse LPS ubiquitylation to antagonize the ubiquitin-dependent anti-bacterial response remains to be elucidated.

The discovery of bacterial LPS as the target of host ubiquitin E3 RNF213 defies the conventional perception that targets of ubiquitylation are proteinaceous and raises the question whether other non-proteinaceous biomolecules, such as host cell lipids also serve as targets of the ubiquitylation machinery. The finding that RNF213-mediated LPS ubiquitylation acts as an initiator of LUBAC-dependent antibacterial autophagy sheds light on the molecular mechanism underlying ubiquitylation-dependent clearance of cytosol-invading bacteria. In addition to LUBAC, LRSAM1, Parkin, and SMURF1 have also been identified as E3 ligases that contribute to antibacterial, autophagy-associated ubiquitylation (Tripathi-Giesgen et al., 2021). It would be intriguing to find out whether and how the connections exist between these E3 enzymes and RNF213. Finally, the details of the mechanism underlying LPS ubiquitylation, such as how the dynein-like ATPase core and the RZ finger contribute to RNF213 function, how LPS is recognized, and precisely which *bona fide* E2 is recruited as RNF213 partner in the cell remain to be elucidated. Answers to these questions may facilitate the decoding of the complex ubiquitin biology and offer insights into ubiquitylation-dependent cell-autonomous defense against cytosolic bacterial invaders.

## References

[B1] Ahel J. Lehner A. Vogel A. Schleiffer A. Meinhart A. Haselbach D. (2020). Moyamoya Disease Factor RNF213 Is a Giant E3 Ligase with a Dynein-like Core and a Distinct Ubiquitin-Transfer Mechanism. Elife 9, e56185. 10.7554/eLife.56185 32573437 PMC7311170

[B2] Kolodziejek A. M. Altura M. A. Fan J. Petersen E. M. Cook M. Brzovic P. S. (2019). Salmonella Translocated Effectors Recruit OSBP1 to the Phagosome to Promote Vacuolar Membrane Integrity. Cel Rep. 27, 2147–2156. 10.1016/j.celrep.2019.04.021 31091452

[B3] Le Negrate G. Faustin B. Welsh K. Loeffler M. Krajewska M. Hasegawa P. (2008). Salmonella Secreted Factor L Deubiquitinase of *Salmonella typhimurium* Inhibits NF-κB, Suppresses IκBα Ubiquitination and Modulates Innate Immune Responses. J. Immunol. 180, 5045–5056. 10.4049/jimmunol.180.7.5045 18354230

[B4] Noad J. Von Der Malsburg A. Pathe C. Michel M. A. Komander D. Randow F. (2017). LUBAC-synthesized Linear Ubiquitin Chains Restrict Cytosol-Invading Bacteria by Activating Autophagy and NF-κB. Nat. Microbiol. 2, 17063. 10.1038/nmicrobiol.2017.63 28481331 PMC5576533

[B5] Otten E. G. Werner E. Crespillo-Casado A. Boyle K. B. Dharamdasani V. Pathe C. (2021). Ubiquitylation of Lipopolysaccharide by RNF213 during Bacterial Infection. Nature 594, 111–116. 10.1038/s41586-021-03566-4 34012115 PMC7610904

[B6] Perrin A. J. Jiang X. Birmingham C. L. So N. S. Y. Brumell J. H. (2004). Recognition of Bacteria in the Cytosol of Mammalian Cells by the Ubiquitin System. Curr. Biol. 14, 806–811. 10.1016/j.cub.2004.04.033 15120074

[B7] Santos J. C. Enninga J. (2016). At the Crossroads: Communication of Bacteria-Containing Vacuoles with Host Organelles. Cell Microbiol. 18, 330–339. 10.1111/cmi.12567 26762760

[B8] Steele S. Radlinski L. Taft-Benz S. Brunton J. Kawula T. H. (2016). Trogocytosis-associated Cell to Cell Spread of Intracellular Bacterial Pathogens. Elife 5, e10625. 10.7554/eLife.10625 26802627 PMC4786427

[B9] Thery F. Martina L. Asselman C. Repo H. Zhang Y. Sedeyn K. (2021). Ring Finger Protein 213 Assembles into a Sensor for ISGylated Proteins with Antimicrobial Activity. Nat. Commun. 12, 5772. 10.1038/s41467-021-26061-w 34599178 PMC8486878

[B10] Tripathi-Giesgen I. Behrends C. Alpi A. F. (2021). The Ubiquitin Ligation Machinery in the Defense against Bacterial Pathogens. EMBO Rep. 22, e52864. 10.15252/embr.202152864 34515402 PMC8567218

[B11] Van Wijk S. J. L. Fricke F. Herhaus L. Gupta J. Hötte K. Pampaloni F. (2017). Linear Ubiquitination of Cytosolic Salmonella Typhimurium Activates NF-κB and Restricts Bacterial Proliferation. Nat. Microbiol. 2, 17066. 10.1038/nmicrobiol.2017.66 28481361

[B12] Wang Y. Yuan S. Jia X. Ge Y. Ling T. Nie M. (2019). Mitochondria-localised ZNFX1 Functions as a dsRNA Sensor to Initiate Antiviral Responses through MAVS. Nat. Cel Biol. 21, 1346–1356. 10.1038/s41556-019-0416-0 31685995

[B13] Whitfield C. Williams D. M. Kelly S. D. (2020). Lipopolysaccharide O-Antigens-Bacterial Glycans Made to Measure. J. Biol. Chem. 295, 10593–10609. 10.1074/jbc.rev120.009402 32424042 PMC7397119

